# Electronic patient-reported outcomes (e-PROMs) in palliative cancer care: a scoping review

**DOI:** 10.1186/s41687-022-00509-z

**Published:** 2022-09-23

**Authors:** Letteria Consolo, Greta Castellini, Silvia Cilluffo, Ilaria Basile, Maura Lusignani

**Affiliations:** 1 Department of Biomedicine and Prevention, University of Rome “Tor Vergata”, Rome, Italy; 2 Bachelor School of Nursing, IRCCS, National Cancer Institute, Milan, Italy; 3 Unit of Clinical Epidemiology, IRCCS Galeazzi Orthopaedic Institute, Milan, Italy; 4 Bachelor School of Nursing, ASST Grande Ospedale Metropolitano Niguarda, University, Milan, Italy; 5 Palliative Care, Pain Therapy and Rehabilitation Unit, IRCCS, National Cancer Institute, Milan, Italy; 6 Associate Professor, Department of Biomedical Sciences for Health, University of Milan, Milan, Italy

**Keywords:** Electronic patient-reported outcome, PROMs, PRO, Self-reported outcome, End of life, Palliative care, Cancer

## Abstract

**Background:**

In palliative oncology settings, electronic patient-reported outcome (PRO) assessment can play an important role in supporting clinical activities for clinicians and patients. This scoping review aims to map the technological innovation of electronic patient-reported outcome measures (e-PROMs) in cancer palliative care and how PRO data collected through e-PROMs can influence the monitoring and management of symptoms and enable better communication between health professionals and patients.

**Methods:**

A scoping review study was designed according to the Arksey and O'Malley framework. Medline, Embase, Web of Science, SCOPUS, PsycINFO and CINAHL and gray literature sources were consulted. The inclusion criteria were people over 18 years old receiving palliative and/or end-of-life care using e-PROMs.

**Results:**

Thirteen primary studies were included: nine quantitative studies, two qualitative studies, and two mixed-method studies. The recently developed software that supports e-PROMs allows patients to receive feedback on their symptoms, helps clinicians prioritize care needs and monitors patients’ conditions as their symptoms change. Electronic PRO data prompt difficult, end-of-life communication between clinicians and patients to better organize care in the last phase of life.

**Conclusion:**

This work shows that electronic PRO data assessment provides valuable tools for patients’ well-being and the management of symptoms; only one study reported conflicting results. However, with studies lacking on how clinicians can use these tools to improve communication with patients, more research is needed.

**Supplementary Information:**

The online version contains supplementary material available at 10.1186/s41687-022-00509-z.

## Background

A patient-centered care (PCC) approach has become the new model guiding today's health care systems [1, 2]. In PCC, patients, relatives and health professionals work together to provide personalized care [3, 4], improving the quality of care [5, 6] and patients’ safety and satisfaction [7, 8]. Patient-centered and quality care also considers how the patients feel and function due to the treatment they receive [9]. Patient-reported outcomes (PROs) provide patients’ perspectives to health care professionals [10]; they are direct reports from patients about their health conditions without interpretation by clinicians [9–11]. PRO data can be collected using standardized questionnaires known as patient-reported outcome measures (PROMs) completed by patients themselves [11–15].

PRO assessment help reduce the gap between clinical realities and patients' wants and needs [16]. Evaluating concepts important to patients and making them an integral part of the care pathway also drives medical team members to set aside time to ensure targeted and personalized care, become more responsive to patients' needs [17–19] and deliver care based on patients' preferences and priorities [20]. Studies show that the systematic use of PRO assessment in routine care supports communication and relationships between health care providers and their patients, making patients feel comfortable enough to detect detailed information about their health status [21].

In clinical practice, PROMs can be in paper or digital format. Today, technology is at the center of daily life. Most people of all ages and backgrounds are comfortable using digital networks or devices, such as touchscreen tablets, smartphones, and computers [22].

The COVID-19 pandemic has encouraged the development of technologies facilitating the remote delivery of health services [23]. The literature shows that technological tools in the use of PROMs have significant advantages over paper tools [13, 22, 24], especially in reducing missing data, resource costs (i.e., monitoring, printing, mailing) and completion time and improving data quality [21, 22]. However, the use of electronic measures presents several challenges, such as training of clinical staff, researchers and patients; overcoming skepticism among health care professionals; and familiarizing patients with electronic devices [25, 26].

A PCC approach is particularly relevant in palliative care [27–29], and PROMs are becoming important tools in this approach [30]. Palliative care is specialized care for patients with an advanced illness to alleviate symptoms and distress caused by the disease itself, seeking to improve the quality of life of patients and caregivers [31].

Palliative cancer patients have to cope with multiple symptoms and complex problems, especially when death is near; poor symptom management has a harmful impact on not only their quality of life but also the use of health care resources [32]. Collection of PRO data allows to determine the effectiveness of a palliative intervention by comparing the health status after the intervention to that before treatment [33–35].

What most people ask for at the end of life, including cancer patients, are management of pain and symptoms, preparation for death, a sense of completion in their lives, and a measure of control in treatment decisions. Patients want a degree of awareness and spiritual peace, completion of funeral arrangements, and the ability to help others while not being a burden [36, 37].

In palliative oncology settings, electronic PRO assessment can play an important role in supporting clinical activities for clinicians and patients [38–40]. Appreciated by patients, e-PROMs in palliative cancer care collect data related to symptoms such as anxiety, drowsiness, fatigue, nausea, and pain and assist clinicians in planning interventions based on symptom severity [39].

This scoping review aims to give a unique overview of the use of electronic PRO data assessment in palliative cancer care. It seeks to map the central concepts in the research identifying the technological innovation of e-PROMs in palliative care and how PRO data collected through e-PROMs can influence the monitoring and management of symptoms and enable better communication between health professionals and their patients.

## Methods

We followed Arksey and O’Malley’s [41] framework and the recommendations by Levac et al. [42]: (1) Identifying the research questions; (2) Identifying relevant studies; (3) Study selection; (4) Charting the data; and (5) Collating, summarizing and reporting the results [41]. The Preferred Reporting Items of Systematic Reviews extension for Scoping Review (PRISMA-ScR) checklist guided the reporting of our scoping review [43]. The protocol was published in the Open Science Framework (OSF) and can be accessed at https://osf.io/3g8tz.

### Stage 1: identifying the research questions

#### Objective

We aimed to map the relevant literature on the use of e-PROMS in palliative care among adult cancer patients.

The following research questions guided the objective:

- What is new in the published, peer-reviewed literature on the technological innovation of e-PROMs in palliative cancer care?
- What is the impact of the PRO data collected by e-PROMs on symptoms' monitoring and management in palliative cancer care?
- How do PRO data collected by e-PROMs support health professional—patient communication in palliative cancer care?

### Stage 2: identifying relevant studies

#### Eligibility criteria

All primary studies were eligible, including those that used either qualitative or quantitative methods with no language and time restriction. The target patient population was people over 18 years of age receiving palliative and/or end-of-life care using e-PROMs. Studies reporting solely on data for the pediatric population were excluded. Studies reporting on both adult and pediatric populations were included only if relevant measures used for the adults were reported separately. Studies on palliative care in a particular stage of cancer care or treatment (i.e., chemotherapy) were considered beyond the scope and thus excluded.

We excluded narrative or systematic reviews and studies for which both abstract and full-text articles were unavailable.

### Stage 3: study selection

#### Information sources

To identify potentially relevant documents, we performed a comprehensive search using the following electronic databases: MEDLINE through Ovid, Embase, Web of Science, SCOPUS, PsycINFO and CINAHL. The search was conducted from inception to July 2022 by two authors. In addition, gray literature searches were carried out using the Google search engine, gray literature databases, and relevant charity and organization websites such as Google Scholar, PsycEXTRA, Open Grey, and OpenThesis.

#### Search strategy

The final search terms included “PROM”, “PRO” “self-reported outcome,” “electronic patient-reported outcome,” “electronic health records,” “e-PROMs,” “end of life,” “palliative care,” “cancer,” and “tumor.” The complete search strategy is provided in the Additional file 1.

#### Selection

The final list of records was transferred for study selection management in the Rayyan Q online reviewing system [44]. For the first screening level, only the titles and abstracts were reviewed to exclude articles that did not meet the inclusion criteria; this work was performed independently by two reviewers to maintain transparency and avoid uncertainty about the outcomes of the review. Titles for which an abstract was not available were not included. The suitability of full-text inclusion was reviewed according to the inclusion and exclusion criteria listed above, and studies that did not meet the inclusion criteria were excluded. In cases of disagreement while selecting studies, we consulted a third reviewer to resolve the dispute and determine the final list of included studies. Cohen's kappa statistic was used to measure the interrater reliability of the study selection [45].

### Stage 4: charting the data

We extracted general characteristics of the included studies, such as year of publication, study location, study population, aims of the study, methodology (quantitative vs. qualitative), types of e-PROMs used, modality of assessment delivery (i.e., software/device used), frequency of e-PROMs used in patient assessment, outcome measures (process of care, assessing patient needs, setting goals, shared decision-making, planning care, outcome monitoring, e-PROMs feedback, intervention reporting frequency, communication effectiveness), notes on usability or satisfaction and supplementary utility.

### Stage 5: collating, summarizing and reporting the results

We collected all the evidence from the included studies by reading them with reference to the three research questions and wrote a narrative summary of the literature about the topic. The studies were analyzed in terms of their general characteristics and with special attention to the e-PROMs that the evidence presented. Frequencies and percentages were utilized to describe nominal data. The results are presented and categorized into four main sections: (1) types of e-PROMs in palliative care; (2) symptoms measured with the different e-PROMs; (3) how these measurements help monitor and manage symptoms and patient care; and (4) how the PRO data collected by e-PROMs add value to patient-clinician communication.

## Results

### Study selection

**Fig. 1:**
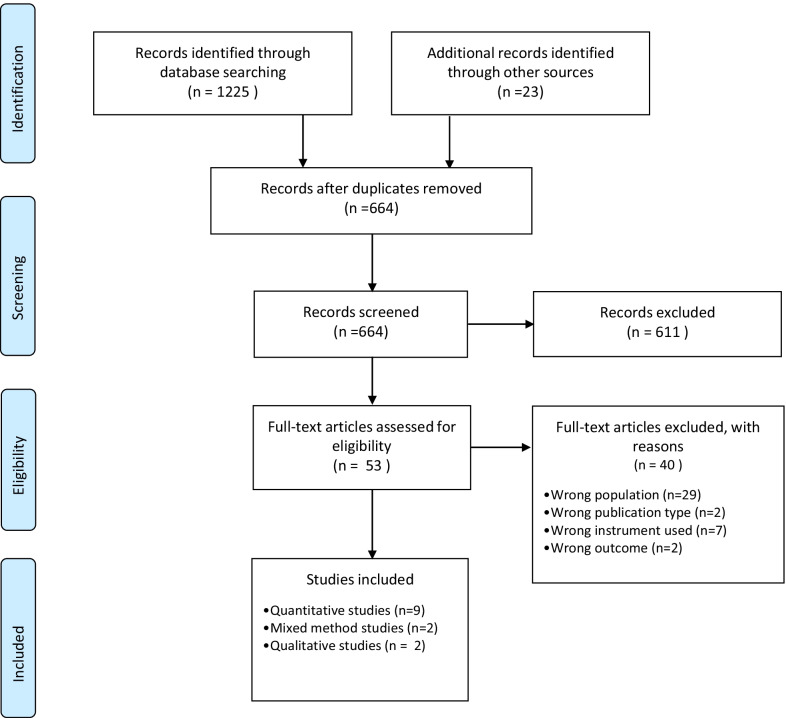
PRISMA flowchart

We found 1248 articles, exported them into Mendeley [46] and screened for duplicates. A total of 584 duplicates were removed, leaving 664 records. Fifty-three articles were found to be eligible, and their full text was read. Articles were excluded if they considered the wrong population (e.g., not palliative care, not oncological care, not terminal care, patient received palliative treatments such as chemotherapy or radiotherapy), had the wrong outcome (i.e., development of PROMs), were the wrong publication type (e.g., a dissertation), or used the wrong tools (e.g., not e-PROMs). The two independent reviewers resolved any disagreements (over, e.g., patients’ characteristics, type of PROMs) through discussion; if consensus could not be reached, a third member was engaged. K was 0.89 with excellent agreement. Thirteen studies were included, of which nine were quantitative [47–54] (one was a protocol used to evaluate new software [55]), two were qualitative [56, 57] and two had a mixed-method design [58, 59]. The search and decision-making process is described using the PRISMA flow diagram [43] in Fig. 1.

### Study characteristics

In Table 1, we report the main characteristics of the included studies.

**Table 1:** Study characteristics

N	Author, year and title	Study design	Sample	Country	Journal of publication
1	Cox et al. (2011) [59] The acceptability of e-technology to monitor and assess patient symptoms following palliative radiotherapy for lung cancer	Mixed-method design	13 clinicians	UK	Palliative Medicine
2	Dy et al. (2011) [47] Tell Us™: A Web-Based Tool for Improving Communication Among patients, Families, and Providers in Hospice and Palliative Care Through Systematic Data Specification, Collection, and Use	Developmental project	HCPS of 3 hospices	USA	Journal of Pain Symptoms Management
3	Giesinger et al. (2011) [52] Quality of Life Trajectory in Patients with Advanced Cancer during the Last Year of Life	Longitudinal study	85 oncology patients	Austria	Journal of Palliative Medicine
4	Suh et al. (2011) [48] Longitudinal patient-reported performance status assessment in the cancer clinic is feasible and prognostic	Longitudinal study	86 patients with inoperable non-small cell lung cancer	USA	Journal of Oncology Practice
5	Hjermstad et al. (2012) [51] Computer-based symptom assessment is feasible in Patients with advanced cancer: Results from an international multicenter study, the EPCRC-CSA	Observational multicenter study	1017 cancer patients	Norway, UK, Austria, Italy, Germany, Switzerland, Canada, Australia	Journal of Pain Symptoms Management
6	Kallen et al. (2012) [58] A technical solution to improving palliative and hospice care	Mixed-method study of usability	27 participants divided into patients, caregivers and providers	USA	Supportive Care in Cancer
7	Stukenborg et al. (2013) [54] Cancer patient-reported outcomes assessment using wireless touch screen tablet computers	Feasibility study through interviews	15 patients with solid tumors	USA	Quality of Life Research
8	LeBlanc et al. (2015) [49] What bothers lung cancer Patient the most? A prospective, longitudinal electronic patient-reported outcomes study in advanced non-small-cell lung cancer	Prospective longitudinal study	97 NSCLC patients	USA	Supportive Care in Cancer
9	Tang et al. (2020) [55] Longitudinal study of symptom burden in outpatients with advanced cancers based on electronic Patient-Reported Outcome (ePRO) platform: a single institution, prospective study protocol	Protocol for a prospective, longitudinal single center cohort study	185 oncology patients	China	BMJ Open
10	Karamanidou et al. (2020) [57] Development of an ePRO based Palliative Care Intervention for Cancer Patients: A Participatory Design Approach	Cross-sectional study employing qualitative methodology via focus group discussions	9 patients with chronic lymphocytic leukemia or myelodysplastic syndromes and 5 HCPs	Greece	Digital Personalized Health and MEDICINE
11	Friis et al. (2021) [53] Patient-Reported Outcome Measures Used in Routine Care Predict for Survival at Disease Progression in Patients Con Advanced lung Cancer	Retrospective explorative study	94 patients with lung cancer	Denmark	Clinical Lung Cancer
12	Bhargava et al. (2021) [50] RELIEF: A Digital Health Tool for the Remote Self-Reporting of Symptoms in Patients with Cancer to Address Palliative Care Needs and Minimize Emergency Department Visits	Pilot study	20 patients in supportive palliative care	Canada	Current Oncology
13	Nipp et al. (2022) [54] Effect of a Symptom Monitoring Intervention for Patients Hospitalized With Advanced Cancer: A Randomized Clinical Trial	Nonblinded randomized clinical trial	321 oncology patients	USA	JAMA Oncology

*UK* United Kingdom, *USA* United States of America, *HCPS* health care professionals, *NSCLC* non-small cell lung carcinoma

The studies were published from 2011 to 2022 and carried out in several European and non-European countries; the most active country publishing on the topic was the USA [47–49, 54, 58].

In seven studies, the e-PROMS were administered through a touchscreen tablet [48, 49, 51–54, 56] and a pen/stylus [51]. Two studies reported the use of smartphones [55, 57], and two used computers [58, 59]. Finally, in two studies, the e-PROMS were usable on any device [47, 50].

Regarding the frequency of use, studies indicated that patients were asked to complete the measurement during visits [48, 49, 51, 53, 56], daily [47, 50, 54, 59], weekly [52, 59] and when indicated by clinicians [55]. The Karamanidou et al. and Kallen et al. studies [57, 58] did not note any required frequency of completion.

Six studies dealt with outpatients [48, 50, 53, 55, 57, 59], five with inpatients [49, 52, 54, 56, 58] and two with both [47, 51].

### Technological innovation of e-PROMs in palliative cancer care

The main characteristics of the software used in the studies and the e-PROMs tools are reported in Table 2.

**Table 2:** e-PROMs characteristics

Software	Device	Characteristics	e-PROMs supported	Domains	Patient outcomes
Tell Us™ Dy et al. [47]	Any	– Opened through standard web browsers, operating systems and hardware platforms; – Can include any questionnaires desired for particular patients; – Feedback through email; – Educational materials	– ND	Functional and psychological symptoms	*Symptoms:* could improve the management of patients' symptoms through questions targeted to their specific needs *Communication:* could improve the quality of information *Supplementary utility:* ND
HealthHUB™ Cox et al. [59]	Computer	– Connected to patients’ landlines at home; – Clinicians receive alerts on their side of the software; – Available symptom-specific advice included in the tool	– ESAS – EQ-5D	Pain, tiredness, nausea, depression, anxiety, drowsiness, appetite, well-being, shortness of breath, mobility, self-care, usual activities, pain/discomfort	*Symptoms:* evaluation, rapid and continuous assessment of symptoms *Communication:* removes eye contact between clinicians and patients, supports patients in expressing their needs *Supplementary utility*: could empower the patient, provide a useful record of patient symptom experience and provide accurate data for audit purposes and commissioner reports
EPCRC-CSA Hjermstadet al. [51]	Tablet	– Each item must be answered before proceeding; – A computerized pain body map is included; – In relation to specific scores, the assessment asks the patient to address other questions more suitable to exploring that problem	– ESAS – EORTC QLQ-C30 – BPI – PRIME-MD PHQ9 – SGA 24	Pain, quality of life, physical function, depression, nutritional intake, need for assistance, time expenditure	*Symptoms:* targeting symptomatic treatment, facilitating symptom assessment. Scores on the EORTC QLQ-C30 were significantly lower among those who did not complete the PROM (*p* < 0.001) and significantly more intense symptoms on the ESAS (*p* = 0.019–0.039) with significantly lower survival (*p* = 0.010) *Communication:* identify areas of particular concern and improve clinician–patient communication *Supplementary utility:* less time spent to complete the assessment than the paper version; elimination of missing responses
MyPAL Karamanidouet al. [57]	Smartphone	– Sends notifications encouraging patients to complete questionnaires; – Contains educational material; – Allow patients to upload photos; – Clinicians can decide to call patients and suggest a visit, exam or therapy;	– ND	– ND	*Symptoms:* viewing symptoms over time could reflect the trajectory of the disease *Communication:* would allow communication on difficult topics with patients *Supplementary utility:* freedom in reporting symptoms; could relieve patients from misinformation and stress
CHES Giesinger et al. [52]	Tablet	– Does not give feedback	– EORTC QLQ-C30	Quality of life	*Symptoms:* Worsening symptoms and are synonymous with disease progression and approaching death and help predict patients’ needs. Physical function decreases significantly in the last 90 days of life (*p* = 0.017), as do sleep disturbances (*p* = 0.039) and taste alterations (*p* = 0.047) *Communication:* standardized self-reports used daily can contribute to symptom management and improve communication *Supplementary utility:* ND
ePROhub Tang et al. [55]	Smartphone	– Runs with the app WeChat; – Clinicians are able to give feedback	– MDASI – ISI – HADS – PHQ-9 – EQ-5D-5L – DT	Insomnia, anxiety and depression, health-related quality of life, mobility, self-care, usual activities, pain/discomfort, distress, practical problems, communication problems, spiritual and religious problems, nausea and vomiting	*Symptoms:* project aims to enable more efficient symptom management at home *Communication:* ND *Supplementary utility:* ND
N.D. Stukenborg et al. [56]	Tablet	– ND	PROMIS questions	Anxiety, depression, fatigue, pain interference, physical function, diarrhea, constipation, nausea, vomiting, anorexia, dyspnea, neuropathy and spiritual values	*Symptoms:* precise measurements improving quality of care *Communication:* improving decision-making by incorporating the patient's perspective *Supplementary utility:* ND
N.D. LeBlanc et al. [49]	Tablet	– ND	PCM version 2.0	Functional and psychological symptoms	*Symptoms:* many items for many different symptoms, more appropriate for patients in disease progression Near death, severe/moderate symptoms increase; 84% of patients have severe fatigue within three months of death (*p* = 0.007) *Communication:* ND *Supplementary utility:* ND
N.D. Suh et al. [48]	Tablet	– ND	PMC 2.0, FACIT-F, FACT-L	Pain, fatigue, nausea, depression, quality of life, insomnia, physical function, appetite/nutritional intake, tiredness, anxiety, drowsiness, emotional problems, well-being, social/role functioning	*Symptoms:* prognostic value of impaired performance (Z = 2.13, *P* = 0.03); has a power similar to KPS and ECOG scores. Impaired ambulation was not statistically significant to evaluate that aspect (Z = − 1.11, *P* = 0.26) *Communication:* ND *Supplementary utility:* performance status metrics recorded by clinicians are buried in text-based documentation, making it difficult to view trends over time. ePRO assessments are reliable, valid, instantly available, and easily tracked longitudinally
N.D. Nipp et al. [54]	Tablet	– ND	– ESAS – PHQ-4	Pain, fatigue, nausea, depression, constipation, appetite/nutritional intake, tiredness, drowsiness, well-being	*Symptoms:* symptom reports provided numeric symptom scores and alerts for any specific symptom worsening by 2 or more points from the previous assessment or for any symptom reaching an absolute score of 4 or higher. They also contained graphs depicting patients’ symptom trajectory. IMPROVED did not have a significant effect on patients’ symptoms or health care use *Communication:* ND *Supplementary utility:* ND
RELIEF Bhargava et al. [50]	Any	– Clinicians are alerted of patients’ score	– ESAS-r – DT – BPI	Pain, fatigue, depression, constipation, drowsiness, tiredness, nausea, appetite loss, anxiety, well-being	*Symptoms:* alert to health care provider if the patients reported an increase of 2 points each day, an increase of 3 points over the previous day, or any score of 8 or higher, for any of the symptoms listed in the ESAS-r, DT, or BPI. RELIEF allowed for timely initiation of appropriate clinical interventions *Communication:* a review of the patient’s care goals was followed by a discussion with the patient and family to discuss the plan to address the symptoms *Supplementary utility*: Reduction of the access to emergency department and admission to hospital, reduction of health care costs, equity in care access
Ambuflex Friis et al. [53]	Tablet	– ND	– EORTC QLQs C30 LC13	Quality of life	*Symptoms:* worsening symptoms give information about prognosis. The absolute value of fatigue (*p* < 0.001) and pain (*p* < 0.001) are indicators of disease progression. Hemoptysis and chest pain offered significant information on survival to progression (*p* < 0.001) *Communication:* helps start end-of-life discussions and aids decision-making by terminally ill patients *Supplementary utility:* ND
N.D. Kallen et al. [58]	Computer	– The provider can access patient records (lab results, PRO assessments, medical history); – Electronic access to an Opioid Converter, a Physician Handbook, and a Nurse Handbook; – The Edmonton Labeled Visual Information System (ELVIS) can be used by health care professionals to document complex cancer burden and treatment information; it demonstrates superior ability over text	– ESAS – CAGE questionnaire	Pain, fatigue, nausea, depression, nutrition intake, tiredness, drowsiness, well-being	*Symptoms:* improve quality of care facilitating temporal and potentially causal relationship between outcomes and clinical events reported on a timeline *Communication:* this system could facilitate communication among members of a multidisciplinary team and patients; help decision-making *Supplementary utility:* improve patients' comprehension of their health status; patients appreciated being able to review and peruse their own medical history and lab results

*ESAS* Edmonton Symptom Assessment System, *EQ-5D* EuroQol 5 dimensions of health, *EORTC QLQ-C30* European Organization for Research and Treatment of Cancer Quality of Life-Core 30, *PRIME-MD PHQ9* Primary Care Evaluation of Mental Disorders nine-item patient health questionnaire, *SGA* Subjective Global Assessment of Nutritional Intake, *MDASI* MD Anderson Symptoms Inventory, *HADS* Hospital Anxiety and Depression Scale, *EQ-5D-5L* EuroQol-5 Dimensions-5 Levels, *PROMIS* Patient-Reported Outcomes Measurement Information System, *PCM* Patient Care Monitor, *EORTC QLQ-C30 LC13* European Organization for Research and Treatment of Cancer Quality of Life-Core 30 Lung Cancer specific, *ISI* Insomnia Severity Index, *FACIT-F* Functional Assessment of Chronic Illness Therapy Fatigue, *FACT-L* Functional Assessment of Cancer Therapy-Lung, *BPI* Brief Pain Inventory, *ESAS-r* Edmonton Symptom Assessment System revised, *DT* distress thermometer, *PHQ-4* four-item patient health questionnaire, *CAGE* Cut Down, Annoyed, Guilty, Eye-opener, *ND* no date

Dy et al. [47] developed the web application Tell Us™, which is available for standard web browsers running on various operating systems and hardware platforms; it can include any questionnaires for particular diagnoses or individual patients. Patients can complete the assessment every day, storing their previous data. This automated software generates an e-mail to the staff for any worrying score, and patients can add comments. Tell Us also provides educational materials [47]. Cox et al. [59] used the computerized software HealthHUB™ for patients in association with CareHUB™ for clinicians. The system sends alerts about patients’ scores on questionnaires daily and weekly; simultaneously, patients have access to symptom-specific advice included in the tool [59]. Hjermstad et al. [51] described the EPCRC-CSA tool, a software application used in various countries with two parts, one for patients and one for clinicians. Inserting a specific score, the assessment asks the patient other questions to explore the problem more deeply [51]. Karamanidou et al. [57] developed MyPAL, a smartphone application that contained educational materials and allowed patients to upload photos. When needed, clinicians could call patients and suggest a visit, exam, or therapy [57]. The software used by Giesinger et al. [52], the computer-based health evaluation system (CHES), does not give feedback and can only collect the assessment data. Tang et al. [55] merged ePROhub software with the app WeChat, which is widely utilized in China. The system automatically recognizes worsening scores and suggests that patients visit the clinic; clinicians can follow patients’ progress and trends in symptom management. Even in the nonblinded randomized clinical trial by Nipp et al., the tool allowed clinicians to depict patients’ symptom trajectory through graphs, with alerts for any symptom worsening by 2 or more points from the previous assessment or for a symptom score of 4 or more [54].

Bhargava et al. presented RELIEF, a digital health tool for the remote self-reporting of symptoms that helps ensure timely clinical intervention by monitoring patients and generating alerts [50].

Kallen et al. designed a prototype software that can be integrated with electronic medical records or serve as a stand-alone product. Clinicians can access patients’ records (e.g., lab results, PRO assessments, medical history) and add notes. The software also supports the Edmonton Labeled Visual Information System (ELVIS) tool, which demonstrates superior ability over text; it can be used by health care professionals to document complex cancer burden and treatment information [58].

### Impact of e-PROMs use on symptom monitoring and management in palliative cancer care

Seventy percent of the assessments were developed to measure the evaluation of symptoms [47, 49, 50, 54–59], and 15% were concerned with quality of life (QoL) [52, 53] or both [48, 51]. Different e-PROMs were used; in particular, four studies used the ESAS scale [51, 54, 58, 59], and three used the EORTC QLQ-C30 [51–53]. The most investigated concepts among patients were pain, fatigue, nausea/vomiting, depression/psychological distress and nutritional problems, as shown in Table 3.

**Table 3:** Concepts most frequently detected in the included studies

Study concepts	Dy et al. [47]	Friis et al. [53]	Giesinger et al. [52]	LeBlanc et al. [49]	Hjermstad et al. [51]	Tang et al. [55]	Karamanidou et al. [57]	Stukenborg et al. [56]	Cox et al. [59]	Suh et al. [48]	Kallen et al. [58]	Nipp et al. [54]	Bhargava et al. [50]	Absolute frequency
Pain			×	×	×	×		×	×	×	×	×	×	10
Fatigue			×	×	×	×		×	×	×	×	×	×	10
Nausea/vomiting			×		×	×		×	×	×	×	×		8
Depression/psychological distress					×	×		×	×	×	×	×	×	8
Nutritional intake/appetite/anorexia			×	×	×			×	×	×	×	×		8
Physical function			×	×	×			×	×	×				6
Tiredness				×	×				×	×	×	×		6
Anxiety				×	×	×		×	×	×				6
Drowsiness					×				×	×	×	×	×	6
Diarrhea/Constipation			×					×				×	×	4
Insomnia/sleep disturbance			×	×		×				×				4
Emotional problems		×	×			×				×				4
QoL		×	×		×					×				4
Well-being										×	×	×		3
Role/social functioning		×	×							×				3
Dyspnea			×					×						2
Neuropathy				×				×						2
Spiritual values						×		×						2
Self-care									×	×				2
Sexual interest				×										1

*QoL* quality of life

× indicates that the concept was measured in the study

Several studies claim that electronic PRO assessment is useful in improving care delivery and the quality of assistance [56], prioritizing and delivering more efficient and effective care [47, 48, 53] and empowering patients to record their own symptoms [54, 57, 59].

Bhargava et al. emphasize that timely initiation of appropriate clinical interventions is enabled by the continuous monitoring of patients’ palliative needs by health care professionals [50].

Multiple studies included in this review underscore how e-PROMs can help with rapid and continuous monitoring of symptoms and predicting the disease’s trajectory [54, 57–59].

LeBlanc et al. [49] and Giesinger et al. [52] showed that in palliative lung cancer patients, fatigue, dyspnea, and insomnia predominate in the last illness stage, with cumulative symptom severity increasing in patients with only three months of life versus > 12 months [49]. Friis et al. [53] showed a longitudinal deterioration of QoL during disease progression, with significant worsening of physical and social functioning (*p* < 0.001), giving a prognostic hypothesis through the onset and worsening of specific symptoms. In the longitudinal study of Suh et al., three measures detected a significant decline in performance: KPS (Z =  − 3.38, *P* = 0.001), ECOG (Z = 3.82, *P* < 0,001), and PMC impaired performance (Z = 2.13, *P* = 0.03); the prognostic value of impaired performance has a power similar to clinician-derived measures when assessed over time. Impaired ambulation was not statistically significant in evaluating that aspect (Z =  − 1.11, *P* = 0.26). In contrast to standard data collected by clinicians, which are often buried in text-based documentation and from which it is difficult to assess trends over time, electronic PRO assessments are continuously updated, and this information is computable, instantly available and easy to track longitudinally [48].

The acceptability and feasibility of electronic PRO assessments are influenced by patients’ physical condition, as shown by Hjermstad et al. and Bhargava et al. [50, 51].

In the multicenter study by Hjermstad et al. [51], 52 of 1017 patients did not complete the full assessment; they reported significantly lower mean scores on the EORTC QLQ C-30 physical functional scale (*p* = 0.001) and received opioid therapy and high scores on the ESAS scale, which means a high symptom burden. As reported by Bhargava et al., 20% of the sample did not complete the assessment because of low function and/or significant fatigue; they reported, as their reasons for nonadherence, having other priorities and the many visits of clinicians. At the same time, even those with high functioning and low symptom burden withdrew from the study because they found the tool repetitive [50].

In three studies, patients preferred the electronic form to the paper form [51, 56, 58].

In Karamanidou et al., patients felt free to report their physical and psychological symptoms, which are often difficult for them to assess [57], through ePROs using MyPAL software.

In the clinical trial of Nipp et al., the symptoms reported via tablet were discussed by the oncology staff every day to identify changes and especially worsening, so all members of the care team were always updated about patients’ symptoms. Nevertheless, this study found no significant intervention effect on the days with improved symptoms, on changes in symptom burden (*P* = 0.17), or on patients’ risk of unplanned readmission (*P* = 0.12) and length of stay (*P* = 0.83) [54].

### The support of e-PROMs in health professional-patient communication in palliative cancer care

The software presented is sufficiently flexible and interactive to improve communication, as noted in most studies [47, 51, 52, 57–59]. It supports the decision-making process [58] by incorporating the patients’ perspectives [56, 57] with feedback systems that automatically alert clinicians if a score is far from a patient’s goal [47, 50, 54, 55, 57].

Most clinicians in the studies by Cox et al. and Kallen et al. [58, 59] felt that standardized tools are beneficial for hospice care. They assist clinical judgment but do not replace face-to-face contact; the clinicians believed that technology should be seen as an addition to in-person encounters from which both clinicians and patients can benefit [59].

The technology also contributes to starting end-of-life discussions, especially as the disease progresses, helping both clinicians and patients become aware of changing perspectives [52, 53] and identifying areas of particular concern to patients or problems that are difficult to discuss [57].

The main characteristics and potential utility of e-PROMs found in the studies selected are reported in Table 4 in the form of the most recurrent expressions in the study texts: “Improve quality and efficacy of palliative care” [47, 48, 52, 53, 56, 59], “Improve communication between patient and providers” [47, 51, 52, 57–59], “Flexible and interactive” [47, 52, 55, 57, 58] and “Support decision-making” [47, 52, 57–59].

**Table 4:** Health professional—patient communication: reported characteristics of e-PROMS in included studies

Study reported characteristics	Dy et al. [47]	Friis et al. [53]	Giesinger et al. [52]	LeBlanc et al. [49]	Hjermstad et al. [51]	Tang et al. [55]	Karamanidou et al. [57]	Stukenborg et al. [56]	Suh et al. [48]	Nipp et al. [54]	Bhargava et al. [50]	Kallen et al. [58]	Cox et al. [59]	Absolute frequency
“Improve quality and efficacy of palliative care”	×	×	×					×	×		×		×	7
“Improve communication between patient and providers”	×		×		×		×					×	×	6
“Flexible and interactive”	×		×			×	×				×	×		6
“Provide feedback”	×						×			×	×	×		5
“Support decision-making”		×	×				×					×	×	5
“Provide alert”	×					×				×	×			4
“Provide more complete picture of individual patient”		×							×			×		3
“Provide educational material”	×						×							2
“Incorporate patients’ prospective/issue of concern”							×	×						2
“Help to initiate end-of-life discussion”		×												1

× indicates that the concept was measured in the study

Furthermore, some studies found that ePRO data assessment has other utilities, such as for audits and commissioner reports [59]. They also noted the shorter time spent completing the ePRO assessment than the paper version and elimination of missing responses [51], less misinformation and stress for patients [57], instant availability and easy longitudinal tracking compared with performance status metrics recorded by clinicians [48], reduced access to emergency departments and hospitals, reduced health care costs, guaranteed equity in care access [48], improved patient comprehension of their health status, and patients’ ability to review their own medical history [58].

## Discussion

This scoping review gives an overview of assessment PRO data using e-PROMs in palliative cancer care. We found thirteen studies published on the topic, describing recently developed software that supports e-PROMs and allows patients to receive feedback on their symptoms, helps clinicians prioritize care needs and monitors the progress of patients’ conditions as their symptoms change.

However, several factors may influence the success of the implementation of electronic PRO data assessment in oncology palliative clinical practice, such as cultural and socioeconomic factors and the e-health literacy and care setting (inpatient vs. outpatient), because patients’ goals and care needs often differ [54].

### Assessment of electronic PRO data

The software was implemented most often on a tablet and administered to patients during hospital visits. Patients used the software to report symptoms such as pain, fatigue, nausea/vomiting, depression/psychological distress and nutritional problems [48–52, 54–56, 58, 59].

Receiving feedback is a good alternative for homebound patients who are very ill and often unable to visit the clinic. They may simply feel more secure with a tool like these; it makes them feel connected to the clinician and not completely alone. Patients truly feel cared for and safe knowing that even if they are far from the hospital, clinicians will always read their data in real time and can give feedback [55].

Electronic PRO data assessment offers advantages over the paper format, appreciated by palliative cancer patients [51, 56, 58] as reported by the state-of-the-art [39]. These types of assessments require a shorter time to complete than the paper version and are considered acceptable by most patients [51, 56]; they also generate fewer missing responses [51], allowing patients to obtain more complete reports of their own health status. Electronic PRO assessments make it easy to assess trends over time and track data longitudinally, in contrast to metrics recorded by clinicians, which are buried in text-based documentation [48].

To become useful tools, an easy-to-use and readily available device should be chosen and the necessary support for its correct use provided. For example, in China, the social network WeChat is used instead of email, as in Western countries [55]. This could be an excellent strategy to achieve greater user compliance and acceptance.

Some aspects to consider in developing the most suitable tools are, for example, the graphic interface; possible visual impairments; unfamiliarity with the technology; using large, clearly visible and understandable characters; and including few icons and buttons on each page to minimum confusion [56]. Another strategy could be presenting only a few items at a time, unlike paper formats, which present all the items on one page at the same time [51].

In addition, electronic PRO data assessment could increase equity in the health care system and ensure high-quality palliative care with no limitations on access due to patients’ geographic location, socioeconomic status, or health care needs [50].

### Symptoms’ management

Using the patient perspective as data is also intended to overcome the supremacy of interventions and decision-making based only on objective data, shifting routine care to a patient-centered approach [59].

Indeed, e-PROMs could also help clinicians estimate prognosis to predict survival to disease progression [49, 53]. The symptoms with most significant deterioration in mean value at disease progression, particularly in patients with advanced lung cancer, are fatigue and pain. The absolute scores of dyspnea, hemoptysis, chest pain and patient-reported performance status offer significant information on survival to progression [48, 49, 52].

The deteriorating health status of these patients is not always synonymous with an inability to use technology; most studies reported that the lowest compliance in the use of electronic devices is found among the sickest patients with progressive health deterioration [47, 49–51, 53]. However, the devices are well accepted, and patients consider them easy to use even if they are unfamiliar with them and require some assistance; indeed, they use them successfully [51, 56, 59]. Compared with patients who completed the entire PROMs, those who did not, report lower scores in physical function [50, 51], which was significant in one study where patients with a low Karnofsky performance status score (40 or less) completed fewer items than those with better performance status [51]. Patients’ clinical conditions must always be taken into account when such assessments are used; patients could be either too sick to use them or too high functioning to see the need for them [50].

Indeed, the use of assessment of PRO that focuses only on the most common symptoms per disease is not recommended, nor is a "one size fits all" approach; efficient and valid assessment promotes the development of personalized care based on the real needs of that individual patient at that precise moment and targeted treatment of symptoms [49, 51].

The final phase of life involves symptoms that have a substantial impact on patients' lives, but only they can actually report which symptoms are the most important and with which they would like help. The determinants of global QOL change toward death; physical functioning becomes less important to patients, whereas the impact of taste alterations, role functioning, and sleep disturbances grows [52].

Electronic PRO data assessment allows real-time reporting of symptoms, which is different from remembering the details of a symptom that occurred days before. This might prevent symptoms being overlooked or underestimated.

Patients can reflect on their symptoms when the software enables them to access their continuous PRO assessment data. This functionality assesses their progress (most of the time worsening), which can help them remain aware of time and of the disease progressing toward certain death, improving their comprehension of their health status [58].

However, the use of electronic PRO data assessment in inpatient cancer palliative care has not always shown statistically relevant results on symptom burden, readmission rates and length of stay [54]. This could suggest that it is still necessary to deepen the impact of technology in that clinical setting.

### Electronic PRO data and communication

These studies have shown how electronic PRO data assessment helps prompt difficult end-of-life communication between clinicians and patients to better organize care in the last phase of life [53]. Objective and subjective data collected through PROMs allow patients to have more informed discussions with their health care providers, particularly helping them know what questions to ask about their own condition [58].

Electronic assessments complement face-to-face interviews without neglecting the previous relationship; almost all of the studies included in this review considered PRO data collected by e-PROMs valuable for improving communication between patients and health care professionals. Of course, electronic devices cannot replace direct contact with professionals, but they represent an additional element to complete and strengthen these relationships.

Communication is crucial, especially during this particular treatment phase. Patients seek reassurance and feedback from clinicians, but often, the little time that clinicians have available for face-to-face meetings is not sufficient to capture all the changes and the occurrence of symptoms over time. In the terminal phase of life, PRO data collected by e-PROMs are effective in improving palliative care, promoting more frequent contact with clinicians, aiding in decision-making, and prioritizing and organizing care during the entire progression of the disease [47, 53, 56–58]. Sometimes questions at this stage of life are uncomfortable and difficult to ask because the answer is not always one that the patient wants to hear.

These tools help empower patients, who increasingly seek control over their illnesses and end-of-life decisions.

## Limitations

This review has several limitations. In performing a scoping review, we attempted to describe all the information available, so we included studies without subjecting them to a formal quality assessment. This work considered a total of thirteen studies from the primary literature, which demonstrates the scarcity of resources available for patients in palliative cancer care who are not subject to any treatments (i.e., palliative chemotherapy or palliative radiotherapy). Most of the included studies were developmental rather than involving patients. There is also a time lag bias due to the COVID-19 pandemic for non-COVID-19-related articles, with a significant increase in submission-to-publication times [60, 61].

## Conclusions

This work has shown that the use of electronic PRO data assessment can be valuable for patients’ well-being and symptom management during palliative care.

Discussion between clinicians and patients can be improved by collaboratively identifying what the patient cares about and needs, helping initiate discussion about the end of life and improving decision-making. Through e-PROMs, clinicians can prioritize patients’ needs according to their questionnaire scores.

Only one study reported conflicting results regarding palliative cancer inpatients and the use of PRO data assessment; this may reflect the need for other studies investigating the use of these data in this setting.

It would be interesting to involve palliative cancer patients at home in studies testing electronic PRO data assessments and even compare the populations of inpatients and outpatients considering the different settings. However, due to the lack of research on this topic, more studies are necessary to better evaluate how clinicians can use electronic PRO data to improve communication with patients.

## Supplementary Information

**Additional file 1.** Complete search strategy for the Medline, Embase, Web of Science, SCOPUS, PsycINFO and CINAHL databases and gray literature. It has been realized in collaboration with a librarian with expertise in systematic searches in medical research databases.

## Data Availability

Data sharing is not applicable to this article, as no datasets were generated or analyzed during the current study. Information about the search strategy is available in the Additional file 1.

## Abbreviations

PRO: Patient-reported outcome
PROM: Patient-reported outcome measure
e-PROM: Electronic patient-reported outcome measure
PCC: Patient-centered care
PRISMA-ScR: Preferred Reporting Items of Systematic Reviews extension for Scoping Review
QoL: Quality of life
ESAS: Edmonton Symptom Assessment System
EQ-5D: EuroQol 5 dimensions of health
EORTC QLQ-C30: European Organization for Research and Treatment of Cancer Quality of Life-Core 30
PRIME-MD PHQ9: Brief Patient Health Questionnaire
SGA: Subjective Global Assessment of Nutritional Intake
MDASI: MD Anderson Symptoms Inventory
HADS: Hospital Anxiety and Depression Scale
EQ-5D-5L: EuroQol-5 Dimensions-5 Levels
PROMIS: Patient-Reported Outcomes Measurement Information System
EORTC QLQ-C30 LC13: European Organization for Research and Treatment of Cancer Quality of life-Core 30 Lung Cancer specific
ISI: Insomnia Severity Index
FACIT-F: Functional Assessment of Chronic Illness Therapy Fatigue
FACT-L: Functional Assessment of Cancer Therapy-Lung
BPI: Brief Pain Inventory
ESAS-r: Edmonton Symptom Assessment System revised
DT: Distress thermometer
PHQ-4: Four-item patient health questionnaire
CAGE: Cut Down, Annoyed, Guilty, Eye-opener
