# Supplementary material for: Electronic patient-reported outcomes (e-PROMs) in palliative cancer care: a scoping review
Source: J Patient Rep Outcomes. 2022 Sep 23;6:102. doi: 10.1186/s41687-022-00509-z (PMC9500127; doi:10.1186/s41687-022-00509-z)
Supplement: Supplementary file 1 — Additional file 1. Complete search strategy for the Medline, Embase, Web of Science, SCOPUS, PsycINFO and CINAHL databases and gray literature. It has been realized in collaboration with a librarian with expertise in systematic searches in medical research databases. [file 41687_2022_509_MOESM1_ESM.docx]

**Data supplement**

**Complete search strategy**

**Ovid MEDLINE(R) <1946 to July Week 3 2022> (273 records retrieved)**

--------------------------------------------------------------------------------

1 (electronic or digital* or computer* or tablet* or smartphone* or software* or app or application* or mobile or web or technol* or informatic* or platform* or ehealth or 'e health' or virtual* or telehealth or 'tele health' or telemedicine or 'tele medicine' or 'tele diagnos*' or telediagnos* or 'tele therap*' or teletherap* or telemonitor* or 'tele monitor*' or teleassess* or 'tele assess*' or internet or remote or online or 'on line' or wireless or 'touch screen' or device* or 'social media' or 'social network*').ab,kf,ti. or exp Electronic Health Records/ or exp Telemedicine/

2 exp Self Report/or exp patient reported outcome measures/ or (pros or proms or prom or selfreport* or 'patient* report*' or 'self report*').ab,kf,ti.

3 (epros or epro or eproms).ab,kf,ti.

4 (1 and 2) or 3

5 exp Cancer exp Neoplasms/

6 exp Medical Oncology/

7 Oncology Nursing/

8 Oncology Service, Hospital/

9 (neoplas* or tumor* or tumour* or cancer* or malignan* or carcinom* or oncol* or 'onco hematol*' or oncohematol* or hematoncol* or hematooncol or 'hemato oncol*').ab,kf,ti.

10 5 or 6 or 7 or 8 or 9

11 Palliative Care/

12 "Hospice and Palliative Care Nursing"/

13 Hospices/

14 exp Terminal Care/

15 exp Terminally Ill/

16 (palliativ* or terminal* or 'end of life' or dying or hospice*).ab,kf,ti.

17 11 or 12 or 13 or 14 or 15 or 16

18 4 and 10 and 17

**Embase (362 records retrieved)**

--------------------------------------------------------------------------------

('electronic patient reported outcome'/exp OR epros:ti,ab,kw OR epro:ti,ab,kw OR eproms:ti,ab,kw OR eprom:ti,ab,kw OR (('patient-reported outcome'/exp OR pros:ti,ab,kw OR proms:ti,ab,kw OR prom:ti,ab,kw OR selfreported:ti,ab,kw OR 'patient* report*':ti,ab,kw OR 'self report*':ti,ab,kw OR 'self report'/exp) AND ('electronic medical record'/exp OR 'electronic health record'/exp OR 'telehealth'/exp OR electronic:ti,ab,kw OR digital*:ti,ab,kw OR computer*:ti,ab,kw OR tablet*:ti,ab,kw OR smartphone*:ti,ab,kw OR software*:ti,ab,kw OR app:ti,ab,kw OR application*:ti,ab,kw OR mobile:ti,ab,kw OR web:ti,ab,kw OR technol*:ti,ab,kw OR informatic*:ti,ab,kw OR platform*:ti,ab,kw OR ehealth:ti,ab,kw OR 'e health':ti,ab,kw OR virtual*:ti,ab,kw OR telehealth:ti,ab,kw OR 'tele health':ti,ab,kw OR telemedicine:ti,ab,kw OR 'tele medicine':ti,ab,kw OR 'tele diagnos*':ti,ab,kw OR telediagnos*:ti,ab,kw OR 'tele therap*':ti,ab,kw OR teletherap*:ti,ab,kw OR telemonitor*:ti,ab,kw OR 'tele monitor*':ti,ab,kw OR teleassess*:ti,ab,kw OR 'tele assess*':ti,ab,kw OR internet:ti,ab,kw OR remote:ti,ab,kw OR online:ti,ab,kw OR 'on line':ti,ab,kw OR wireless:ti,ab,kw OR 'touch screen':ti,ab,kw OR device*:ti,ab,kw OR 'social media':ti,ab,kw OR 'social network*':ti,ab,kw))) AND ('cancer pain'/exp OR 'cancer patient'/exp OR 'neoplasm'/exp OR 'oncology'/exp OR 'oncology nursing'/de OR 'oncology ward'/exp OR neoplas*:ti,ab,kw OR tumor*:ti,ab,kw OR tumour*:ti,ab,kw OR cancer*:ti,ab,kw OR malignan*:ti,ab,kw OR carcinom*:ti,ab,kw OR oncol*:ti,ab,kw OR 'onco hematol*':ti,ab,kw OR oncohematol*:ti,ab,kw OR hematoncol*:ti,ab,kw OR hematooncol:ti,ab,kw OR 'hemato oncol*':ti,ab,kw) AND ('palliative therapy'/exp OR 'palliative nursing'/exp OR 'hospice'/exp OR 'hospice nursing'/exp OR 'terminal care'/exp OR 'terminally ill patient'/exp OR palliativ*:ti,ab,kw OR terminal*:ti,ab,kw OR 'end of life':ti,ab,kw OR dying:ti,ab,kw OR hospice*:ti,ab,kw) AND ('article'/it OR 'article in press'/it OR 'conference paper'/it OR 'preprint'/it OR 'review'/it)

**PsycINFO <1806 to July Week 3 2022> (69 records retrieved)**

--------------------------------------------------------------------------------

1 exp Patient Reported Outcome Measures/

2 (pros or proms or prom or selfreport* or 'patient* report*' or 'self report*').ab,id,ti.

3 1 or 2

4 exp Electronic Health Records/

5 exp Telemedicine/

6 (electronic or digital* or computer* or tablet* or smartphone* or software* or app or application* or mobile or web or technol* or informatic* or platform* or ehealth or 'e health' or virtual* or telehealth or 'tele health' or telemedicine or 'tele medicine' or 'tele diagnos*' or telediagnos* or 'tele therap*' or teletherap* or telemonitor* or 'tele monitor*' or teleassess* or 'tele assess*' or internet or remote or online or 'on line' or wireless or 'touch screen' or device* or 'social media' or 'social network*').ab,id,ti.

7 4 or 5 or 6

8 exp palliative care/

9 exp Hospice/

10 (palliativ* or terminal* or 'end of life' or dying or hospice*).ab,id,ti.

11 exp Terminally Ill Patients/s/ or exp "Death and Dying"/

12 8 or 9 or 10 or 11

13 (neoplas* or tumor* or tumour* or cancer* or malignan* or carcinom* or oncol* or 'onco hematol*' or oncohematol* or hematoncol* or hematooncol or 'hemato oncol*').ab,id,ti.

14 exp Neoplasms/

15 exp Oncology/

16 13 or 14 or 15

17 12 and 16

18 exp Terminal Cancer/

19 17 or 18

20 3 and 7 23548

21 (epros or epro or eproms or eprom).ab,id,ti. 42

22 20 or 21 23550

23 19 and 22 69

**CINAHL (231 records retrieved)**

--------------------------------------------------------------------------------

| S18 | S11 AND S16 AND S17 |
| --- | --- |
| S17 | S14 OR S15 |
| S16 | S12 OR S13 |
| S15 | TI (neoplas* or tumor* or tumour* or cancer* or malignan* or carcinom* or oncol* or 'onco hematol*' or oncohematol* or hematoncol* or hematooncol or 'hemato oncol*') OR AB (neoplas* or tumor* or tumour* or cancer* or malignan* or carcinom* or oncol* or 'onco hematol*' or oncohematol* or hematoncol* or hematooncol or 'hemato oncol*') |
| S14 | (MH "Neoplasms+") OR (MH "Cancer Patients") OR (MH "Oncologic Nursing") OR (MH "Radiation Oncology Nursing") OR (MH "Oncologic Care") OR (MH "Oncology Care Units") |
| S13 | TI ((palliativ* or terminal* or 'end of life' or dying or hospice*)) OR AB ((palliativ* or terminal* or 'end of life' or dying or hospice*)) |
| S12 | (MH "Palliative Care") OR (MH "Palliative Medicine") OR (MH "Hospices") OR (MH "Hospice Care") OR (MH "Hospice Patients") OR (MH "Terminal Care+") OR (MH "Terminally Ill Patients+") |
| S11 | S9 OR S10 |
| S10 | TI (EPROS OR EPRO OR EPROMS EPROM) OR AB (EPROS OR EPRO OR EPROMS EPROM) |
| S9 | S4 AND S8 |
| S8 | S5 OR S6 OR S7 |
| S7 | TI (electronic or digital* or computer* or tablet* or smartphone* or software* or app or application* or mobile or web or technol* or informatic* or platform* or ehealth or 'e health' or virtual* or telehealth or 'tele health' or telemedicine or 'tele medicine' or 'tele diagnos*' or telediagnos* or 'tele therap*' or teletherap* or telemonitor* or 'tele monitor*' or teleassess* or 'tele assess*' or internet or remote or online or 'on line' or wireless or 'touch screen' or device* or 'social media' or 'social network*') OR AB (electronic or digital* or computer* or tablet* or smartphone* or software* or app or application* or mobile or web or technol* or informatic* or platform* or ehealth or 'e health' or virtual* or telehealth or 'tele health' or telemedicine or 'tele medicine' or 'tele diagnos*' or telediagnos* or 'tele therap*' or teletherap* or telemonitor* or 'tele monitor*' or teleassess* or 'tele assess*' or internet or remote or online or 'on line' or wireless or 'touch screen' or device* or 'social media' or 'social network*') |
| S6 | (MH "Telehealth+") |
| S5 | (MH "Electronic Health Records+") |
| S4 | S1 OR S2 OR S3 |
| S3 | (MH "Self Report+") |
| S2 | TI (pros or proms or prom or selfreport* or patient-report* or self-report*) OR AB (pros or proms or prom or selfreport* or "patient report*" or "self report*") |
| S1 | (MH "Patient-Reported Outcomes+") |
|  |  |

**Scopus (161 items retrieved)**

--------------------------------------------------------------------------------

(((TITLE-ABS-KEY ("Patient Reported Outcome*" OR proms OR "patient-reported treatment outcome" OR "patientreported outcome" OR "self-reported outcome" OR "self-reported patient outcome" OR "self-reported treatment outcome" OR "selfreported outcome") AND TITLE-ABS-KEY (electronic OR digital OR computer OR tablet OR smartphone* OR software* OR app OR application* OR mobile))) OR (TITLE-ABS-KEY (epros OR epro OR eproms AND eprom))) AND (TITLE-ABS-KEY (neoplas* OR tumor* OR tumour* OR cancer* OR malignan* OR carcinom* OR oncol* OR onco-hematol* OR oncohematol* OR hematoncol* OR hematooncol OR hemato-oncol*)) AND (TITLE-ABS-KEY (palliativ* OR terminal* OR "END OF LIFE" OR dying OR hospice*))

**Web of Science (129 records retrieved)**

--------------------------------------------------------------------------------

#3 AND #4 AND #5

5 PALLIATIV* OR TERMINAL* OR “END OF LIFE” OR DYING or hospice* (Topic)

4 TS=(Neoplas* OR Tumor* OR TUMOUR* OR Cancer* OR Malignan* OR carcinom* OR ONCOL* OR ONCO-HEMATOL* OR ONCOHEMATOL* OR HEMATONCOL* OR HEMATOONCOL OR HEMATO-ONCOL*)

3 #1 OR #2

2 EPROS OR EPRO OR EPROMS EPROM (Topic)

1 TS=(“Patient Reported Outcome*” OR PROMS OR “patient-reported treatment outcome” OR “patientreported outcome” OR “self-reported outcome” OR “self-reported patient outcome” OR “self-reported treatment outcome” OR “selfreported outcome”) AND TS=(ELECTRONIC OR DIGITAL OR COMPUTER OR TABLET OR SMARTPHONE* OR SOFTWARE* OR APP OR APPLICATION* OR MOBILE)

**Google Scholar/APA PsycExtra/Open Grey/OpenThesis (23 records retrieved)**

--------------------------------------------------------------------------------

(palliative) AND (oncology) AND (ePROMs or "electronic patient reported outcomes)
